# Heat-mediated manipulation of gene expression by IR-LEGO in the developing genitalia in *Drosophila*

**DOI:** 10.1093/g3journal/jkag035

**Published:** 2026-02-12

**Authors:** Moe Onuma, Tatsuyuki Kumagai, Kentaro Hayashi, Yasuhiro Kamei, Aya Takahashi

**Affiliations:** Department of Biological Sciences, Tokyo Metropolitan University, Hachioji 192-0397, Japan; Department of Biological Sciences, Tokyo Metropolitan University, Hachioji 192-0397, Japan; Optics and Imaging Facility, Trans-Scale Biology Center, National Institute for Basic Biology, Okazaki 444-8585, Japan; Optics and Imaging Facility, Trans-Scale Biology Center, National Institute for Basic Biology, Okazaki 444-8585, Japan; Basic Biology Program, The Graduate University for Advanced Studies (SOKENDAI), Okazaki 444-8585, Japan; Department of Biological Sciences, Tokyo Metropolitan University, Hachioji 192-0397, Japan; Research Center for Genomics and Bioinformatics, Tokyo Metropolitan University, Hachioji 192-0397, Japan

**Keywords:** infrared laser-evoked gene operator system (IR-LEGO), heat shock, RNAi, *Drosophila*, genitalia

## Abstract

Manipulating gene expression in a tissue-specific and temporally controlled manner is essential for understanding the function of the focal genes. Still, in many cases, the limited availability of specific promoters to drive ectopic manipulation remains a restricting factor in developing organs, even in *Drosophila*. Developing external genitalia is one such organ with a complex anatomical structure shaped by a joint regulatory network of many transcription factors. To overcome the restriction, we employed the infrared laser-evoked gene operator system (IR-LEGO), in which infrared laser (1,480 nm) irradiation induces gene expression under the control of a heat shock promoter. Pupal genital structures were irradiated at approximately 24 or 48 h after puparium formation. We tested a range of laser power and depth to the target structure by a reporter assay using green fluorescent protein, which was induced under the control of the *heat shock protein 70* promoter (*hs*-*GAL4*). In previous studies, the IR-LEGO has been used as a tool to induce ectopic transgene expression. In this study, we attempted to knock down genes such as *yellow* (*y*) and *odd-paired* (*opa*) ectopically by RNAi using the GAL4/UAS system. The results demonstrated that this technique has a high potential in manipulating transcript abundance levels in small groups of cells in specific genital structures to unravel novel functions of genes involved in the morphogenesis of species-specific and rapidly evolving anatomical structures.

## Introduction

Manipulating gene expression is essential for understanding the functions of the focal genes. In particular, regulating gene expression in a tissue-specific and temporally controlled manner is often required to overcome complexity caused by pleiotropic effects from multiple expression domains of the focal genes. Various genetic tools have been developed to achieve this objective. A groundbreaking technique, the GAL4/UAS system utilizing the yeast galactose-inducible system has proven to be versatile and highly effective in manipulating transcription activation in *Drosophila melanogaster* (Brand and Perrimon 1993) and has been widely used. It can also be used in a slightly limited manner in mice and zebrafish (Asakawa and Kawakami 2008; Halpern et al. 2008). However, despite its widespread application, finding the correct GAL4 driver for a specific purpose is not trivial when the drivers that are activated at a specific time and space of interest are not available due to pleiotropy. Further methodological refinement is necessary to conduct precise spatial and temporal manipulation of the gene expression levels in such cases.

External genitalia, especially that of males, is one of the most complicated and rapidly diversifying anatomical structures among animal body parts (Eberhard 1985; Hosken and Stockley 2004; Simmons 2014). Recent studies using pairs of closely related *Drosophila* species have highlighted the expression diversifications of genes that are responsible for interspecific morphological differences in specific genital structures (Nagy et al. 2018; Hagen et al. 2019; Ridgway et al. 2024). However, the molecular basis of the unique morphological evolution of genital structures remains largely elusive.

The whole terminalia (genitalia and analia) develops from the genital imaginal disc, and a higher-order morphogenesis takes place throughout pupal development. While genetic regulation in the imaginal discs has been described in more detail (Gorfinkiel et al. 1999; Keisman and Baker 2001; Chatterjee et al. 2011; Ridgway et al. 2024), a high-throughput in situ hybridization assay of the pupal terminalia (including genital and anal structures) of male *D. melanogaster* has depicted the expression landscapes of over 100 transcription factors (Vincent et al. 2019). Also, the morphogenesis anatomy of male pupal terminalia in 12 *Drosophila* species with diverse morphology has been described and compared in detail (Urum et al. 2024; Urum and Preger-Ben Noon 2025). The functional assays of the transcription factors and other genes would apparently be the next step in tackling the question of how gene regulatory networks shape complex morphogenesis and facilitate diversification. Such an approach requires precise manipulation of gene expression at specific substructures or cell populations at specific timings. The GAL4 driver commonly used for the male genital disc, *NP6333-GAL4*, also drives expression in wing and leg discs (Stieper et al. 2008). Other *enhancer-*GAL4 drivers for multiple genes, including *apterous*, *Pox neuro*, and others (eg *eyes absent*, *empty spiracles*, and *Gef64C*), have been shown to drive expression in subsets of developing male genitalia (Boll and Noll 2002; Frazee and Masly 2015; Glassford et al. 2015). However, because these genes typically have multiple pleiotropic roles during development, manipulating gene expression in restricted domains of developing genitalia in vivo is still challenging. Motivated by this demand, we have developed a heat-mediated manipulation system of gene expression in the developing genitalia of *D. melanogaster*.

In this study, we employed the infrared laser-evoked gene operator system (IR-LEGO, Kamei et al. 2009), in which infrared laser light irradiation induces gene expression under the control of a heat shock promoter. The method has been successfully applied to *Caenorhabditis elegans* (Kamei et al. 2009; Suzuki et al. 2013, 2022), *Danio rerio* (Deguchi et al. 2009; Kimura et al. 2013), *Oryzias latipes* (Deguchi et al. 2009; Kobayashi et al. 2013; Okuyama et al. 2013; Shimada et al. 2013), *Xenopus laevis* (Kawasumi-Kita et al. 2015; Hasugata et al. 2018), *Pleurodeles waltl* (Kawasumi-Kita et al. 2015), *Arabidopsis thaliana* (Deguchi et al. 2009; Hwang et al. 2019; Tomoi et al. 2023), *Physcomitrium patens* (Tomoi et al. 2024), *Daphnia magna* (Shimizu et al. 2024), and *D. melanogaster* (Miao and Hayashi 2015). In this study, we combined the inducible RNAi knockdown system with IR-LEGO for the first time.

In brief, the pupal terminalia at approximately 24 or 48 h after puparium formation (h APF) were irradiated by an infrared laser. The efficiency of the heat shock induction was examined using *hs-GAL4*/*UAS-GFP* flies, in which the green fluorescent protein (GFP) is induced under the control of the *heat shock protein 70* promoter. Furthermore, we knocked down the *y* and *opa* genes by the inducible RNAi system. We demonstrate that the technique is promising in manipulating transcript abundance in small groups of cells in specific genital structures and can be employed to unravel novel functions of genes in the morphogenesis of rapidly evolving genital structures.

## Materials and methods

### Fly stocks

The following *D. melanogaster* strains were used in the present study: *hs-GAL4* (#106-509) from the Kyoto *Drosophila* Stock Center, *UAS-CD4-tdGFP* (#35838) from the Bloomington *Drosophila* Stock Center, *UAS-opa-RNAi* (v101531) from the Vienna *Drosophila* Resource Center, *UAS-y*-*RNAi* (3757R-1) and *UAS-opa*-*RNAi* (1133R-3) from the National Institute of Genetics. The NIG-Fly assessment of phenotypes induced by *Act5C*-*GAL4* at 28 °C showed “yellow body color” and “lethal” for 3757R-1 and 1133R-3, respectively, indicating sufficient efficiency of RNAi knockdown. *NP6333-GAL4* (*P{GawB}Pen^NP6333^*) carrying the *UAS-Dicer-2* transgene (*P{UAS-Dcr-2.D}*) (Chatterjee et al. 2011) was used as a disc-wide GAL4 driver. All flies were maintained under the 12-h light–dark condition at 25 ± 1 °C on the standard corn medium.

### Sample preparation

**Fig. 1.**
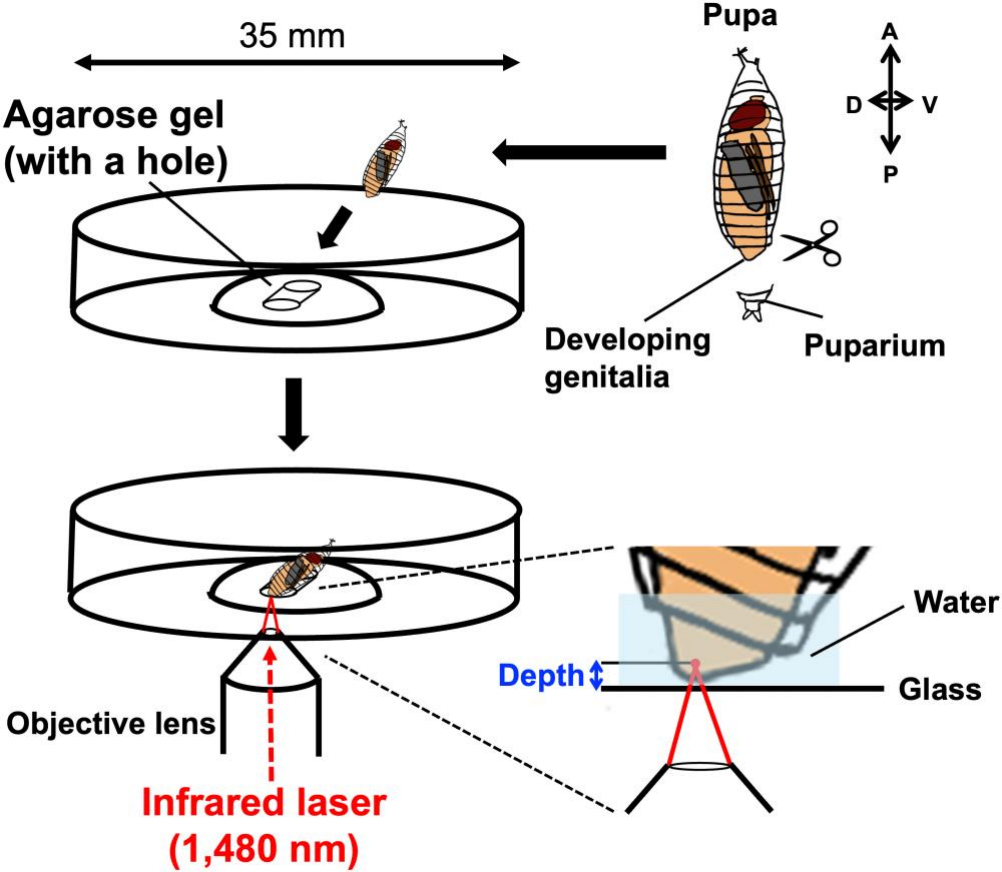
Sample preparation and laser irradiation of the pupal terminalia. The puparium at the abdominal tip was removed to expose the developing genitalia. The pupa was placed onto a glass-bottom petri dish through an angled hole made in a drop of solidified agarose. An infrared laser (1,480 nm) was applied through the objective lens and irradiated the target cells at a certain distance (depth) from the glass.

Male and female pupae were collected at the white pupal stage by sorting gonad size and placed in a humid chamber at 25 °C. The puparium at the abdominal tip was removed by fine forceps at 24 ± 1 h APF or 48 ± 1 h APF and placed onto a glass-bottom petri dish (35-mm dish diameter, 14-mm glass diameter, glass thickness 0.16 to 0.19 mm; D11130H, Matsunami, Japan) through a hole made by 1,000-uL pipetman tip to a 250- to 300-μL drop of solidified 1.5% agarose (Fig. 1). The hole needs to be tilted to allow light penetration through the tissues to visualize the target structures. Distilled water was supplied to fill the space between the specimen and the glass.

### IR-LEGO system

An inverted microscope (IX-81, Evident, Japan) was equipped with an IR-LEGO optical unit (custom assembled; Sigma Koki, Japan). An objective lens, UApo340 20× (NA = 0.75 UV) (custom-made; Evident, Japan), was used to visualize the target tissues and focus irradiation with an infrared laser (1,480 nm). The distance between the bottom glass (marked by an ink pen) and the focal cells (Fig. 1) was measured using the images captured by a charge-coupled device (CCD) camera (ORCA-Flash 4.0, C11440, Hamamatsu Photonics, Japan) attached to the inverted microscope. MetaMorph Software (Molecular Devices, Japan) was used to measure the distance (depth). The laser power (dBm) was calibrated by using a Head Sensor for Laser Power Meter 10A (Ophir 7Z02637, Japan) and a Vega Laser Power and Energy Meter (Ophir 7Z01560, Japan). A 6- to 8-mW laser through the objective lens was emitted to heat the live specimen for 60 s.

### Observation of fluorescent signal and morphology after irradiation

Irradiated pupa was kept in the agarose gel inside the petri dish with the lid closed to avoid desiccation. The petri dish was placed at 25 °C until eclosion. For the *hs*-*GAL4*/*UAS-CD4-tdGFP* individuals, the fluorescent signal was observed on the following day (14 to 24 h after irradiation) by a band path filter set (470- to 495-nm excitation filter, 510- to 550-nm emission filter, 505-nm dichroic mirror) equipped with an inverted microscope (IX-81, Evident, Japan). Fluorescent images were taken with a CCD camera (ORCA-Flash 4.0, C11440, Hamamatsu Photonics, Japan) attached to the microscope.

Adults that emerged from the pupae after irradiation were taken out of the petri dish and placed into 70% ethanol at 6 to 48 h after eclosion. The *hs*-*GAL4*/*UAS*-*y*-*RNAi* and the *hs*-*GAL4*/*UAS*-*opa*-*RNAi* pupae were dissected in 70% ethanol, and the periphallic genital organs of males or the hypogyniums of females were mounted in 50% Hoyer's solution (Hoyer's medium: acetic acid = 1:1) and incubated at 60 °C overnight. Images of genital organs were captured by a CCD camera (DP73, Evident, Japan) attached to an inverted microscope (IX73, Evident, Japan). In addition, genitalia images of the samples subjected to *NP6333-GAL4* driven RNAi treatments were also conducted by the procedure described above.

### Linearity scores of the proximal bristle alignment on the surstylus

The linearity of the 6 most proximal bristles on the surstylus was evaluated by the proportion of variance explained by the first principal component (PC1) extracted by the principal component analysis (PCA). PCA was performed using the PCA implementation in the scikit-learn Python library (Pedregosa et al. 2011). The bases of the bristles surrounded by a socket cell on the photo images were manually marked, and their X and Y coordinates were obtained using Fiji (Schindelin et al. 2012). The PC1 of the coordinates represents a line that minimizes perpendicular distances from all points. The proportion of the variance explained by this component is an angle-independent measure of linearity. Therefore, bristle coordinates with a score closer to 1 are more linearly aligned.

## Results

### Optimal IR-LEGO conditions for developing pupal genitalia in *Drosophila*

The optimal condition to induce heat shock-mediated gene expression varies among organisms and possibly among genetic systems employed (Deguchi et al. 2009). The laser power, irradiation duration, and the distance between the cover glass and the focal cells (hereafter depth) are the factors affecting the efficiency because of the attenuation of laser power due to the absorption of water in the tissue or gel in front of the focal cells. Previous investigations have shown that longer irradiation (ie 60 s) results in a more stable heat shock response compared to shorter durations, and a higher laser power will have a higher risk of damaging the cell (Kamei et al. 2009; Tomoi et al. 2024). Thus, for the developing pupal genitalia, we tested 6-, 7-, and 8-mW laser power with a duration of 60 s, targeting cells at various depths.

**Fig. 2.**
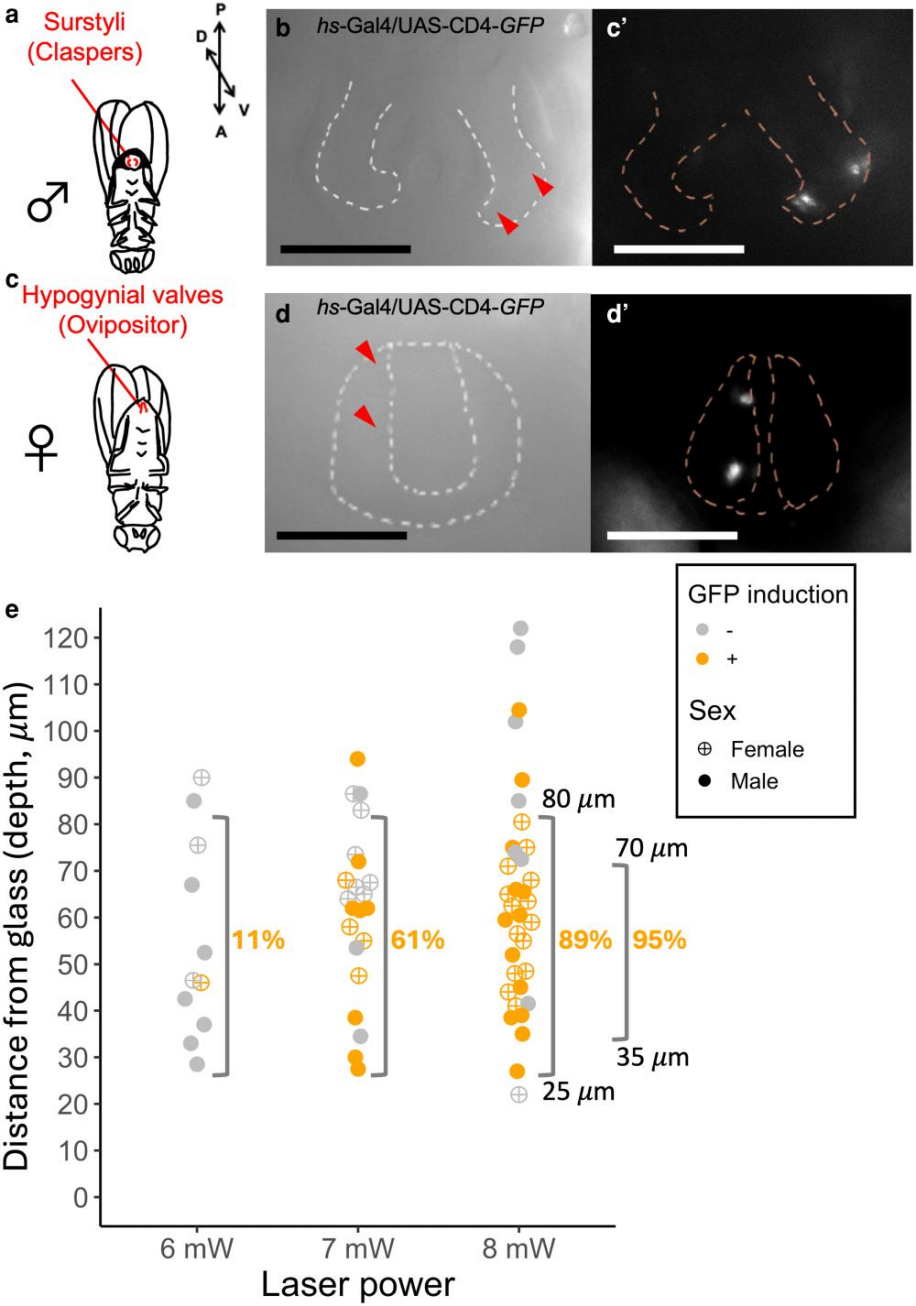
Testing experimental conditions for IR-LEGO in *Drosophila* pupae. a) Surstyli of an adult male. b) Bright-field image of the irradiated surstylus (outlined by a dotted line) of a male *hs-GAL4/UAS-CD4-tdGFP* at 48 ± 1 h APF. The arrowheads indicate the approximate location of the 2 irradiated positions by an 8-mW laser for 60 s. b’) GFP signal of the pupal genitalia captured 23.5 h after infrared laser irradiation to the surstylus. Outlines of the pair of surstyli are indicated by dotted lines. c) Hypogynial valves in an adult female. d) Bright-field image of the irradiated hypogynial valve (outlined by a dotted line) of a female *hs-GAL4/UAS-CD4-tdGFP* at 48 ± 1 h APF. The arrowheads indicate the approximate location of the 2 irradiated positions by an 8-mW laser for 60 s. d’) GFP signal of the pupal genitalia captured 21.5 h after infrared laser irradiation to the hypogynial valve. Outlines of the pair of valves are indicated by dotted lines. e) Presence and absence of GFP signal assessed 14 to 24 h after irradiation by a 6-, 7-, and 8-mW laser in the target cells at various distances (depths) from the glass. Percentages of GFP-positive samples are shown within the ranges indicated by brackets. Scale bars indicate 100 μm.

To find the optimal condition, the pupal genitalia of *hs-GAL4*/*UAS-CD4-tdGFP* at 48 ± 1 h APF, when structures of male and female external genitalia such as surstylus (clasper) (Rice et al. 2019) and hypogynial valve (ovipositor) (McQueen et al. 2022) become visible (Glassford et al. 2015; Smith et al. 2020; Urum et al. 2024), were targeted. The laser was applied to 2 well-focused positions (chosen arbitrarily) on the right side of the surstylus (for a male, Fig. 2a) or the hypogynial valve (for a female, Fig. 2b), leaving the other side intact. The GFP signal was visible the next day when the heat shock-mediated GFP induction was successful (Fig. 2, b to b’ and d to d’).

The depth of the target structure could not be precisely controlled due to the variation in the shape of the puparium. Therefore, we tested the responses of the target groups of cells at various depths using different laser power settings. The results are shown in Fig. 2e and Supplementary Table 1. The minimum depth, which was restricted by the pupal cuticle covering the developing structures, among the samples was 22 μm. Out of 68 samples tested, 40 showed GFP signals. Of those GFP-positive samples, 36 (90%) exhibited positive signals at 2 distinct positions, including 8 samples (20%) that showed continuous, nonseparable signals between the 2 positions. The samples with at least 1 detectable signal were scored as GFP-positive. The results indicated that GFP signal was detected in 89% of the samples within the depth range 25 to 80 μm when irradiated using an 8-mW infrared laser beam for 60 s. The success rate increased to 95% when the depth range was confined to 35 to 70 μm. The eclosion rate of the irradiated samples was 91.9% (*N* = 74). According to these results, the samples with depth > 80 μm were discarded in the following experiments.

### The heat-mediated RNAi knockdown of the *y* gene in small groups of cells

**Fig. 3.**
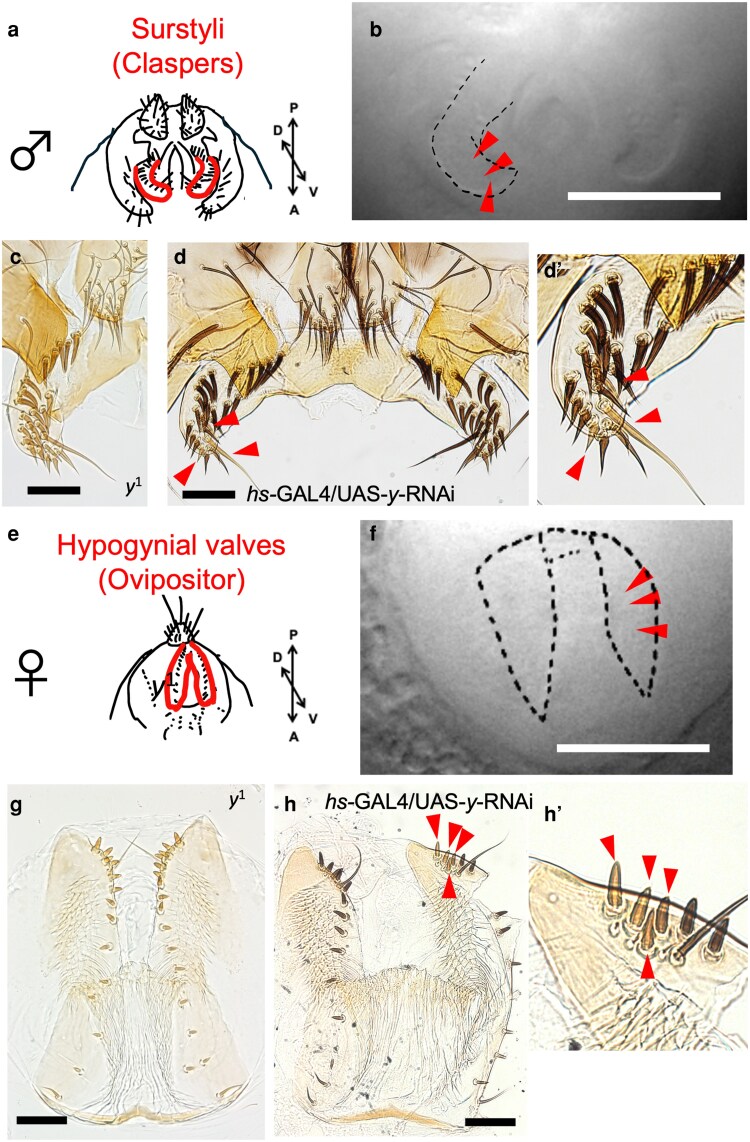
RNAi knockdown of *y* by IR-LEGO in pupal genitalia. a) Surstyli of an adult male. b) Bright-field image of the irradiated surstylus (outlined by dotted line) at 48 ± 1 h APF. The arrowheads indicate the approximate location of the 3 irradiated positions by an 8-mW laser for 60 s. c) Surstylus of *y*^1^ mutant. d) Left (irradiated) and right (intact) surstyli of an *hs-GAL4*/*UAS-y-RNAi* male after eclosion. d’) Magnified image of the left surstylus. The arrowheads indicate the bristles with reduced black pigmentation. e) Hypogynial valves of an adult female. f) Bright-field image of the irradiated valve, with outlines of left and right valves indicated by dotted lines, at 48 ± 1 h APF. The arrowheads indicate the approximate location of the 3 irradiated positions by an 8-mW laser for 60 s. g) Hypogynial valves of *y*^1^ mutant. h) Left (intact) and right (irradiated) valves of an *hs-GAL4*/*UAS-y-RNAi* female after eclosion. h’) Magnified image of the right valve. The arrowheads indicate the bristles with reduced black pigmentation. Scale bars indicate 100 μm.

Since the RNAi knockdown by IR-LEGO has not been conducted before, we tested the effect of heat-mediated knockdown using the *y* gene, whose classic mutants exhibit yellow body color due to the lack of an ability to synthesize dark-colored pigments (Fig. 3, c and g). We used the same GAL4/UAS system as in the case of the GFP reporter assay (Fig. 2), and the *hs-GAL4*/*UAS-y-RNAi* individuals were subjected to infrared laser irradiation targeting 1 side of the paired surstyli (Fig. 3, a and b) or hypogynial valves (Fig. 3, e and f). Three positions close to the tip of those structures at 48 ± 1 h APF (Fig. 3, b and f) were irradiated by an 8-mW infrared laser beam for 60 s (Supplementary Table 2). After eclosion, a small number of yellow-colored bristles, like those of *y*^1^, were observed only on the irradiated sides of those structures (Fig. 3, d to d’ and h to h’). All 10 individuals (5 females and 5 males) subjected to this treatment showed changes in bristle pigmentation intensity on the irradiated side, although the magnitude of change was small in 2 male samples (Supplementary Table 2). This phenotype was not observed in male and female individuals in the GFP reporter assay subjected to the same irradiation treatment and showed a clear GFP signal (Supplementary Figs. 1 and 2). These results indicated that infrared light irradiation can be used to induce RNAi knockdown by the heat-mediated inverted repeat RNA transcription.

### The heat-mediated RNAi knockdown of the *opa* gene at the border region of the epandrial ventral lobe and surstylus

**Fig. 4.**
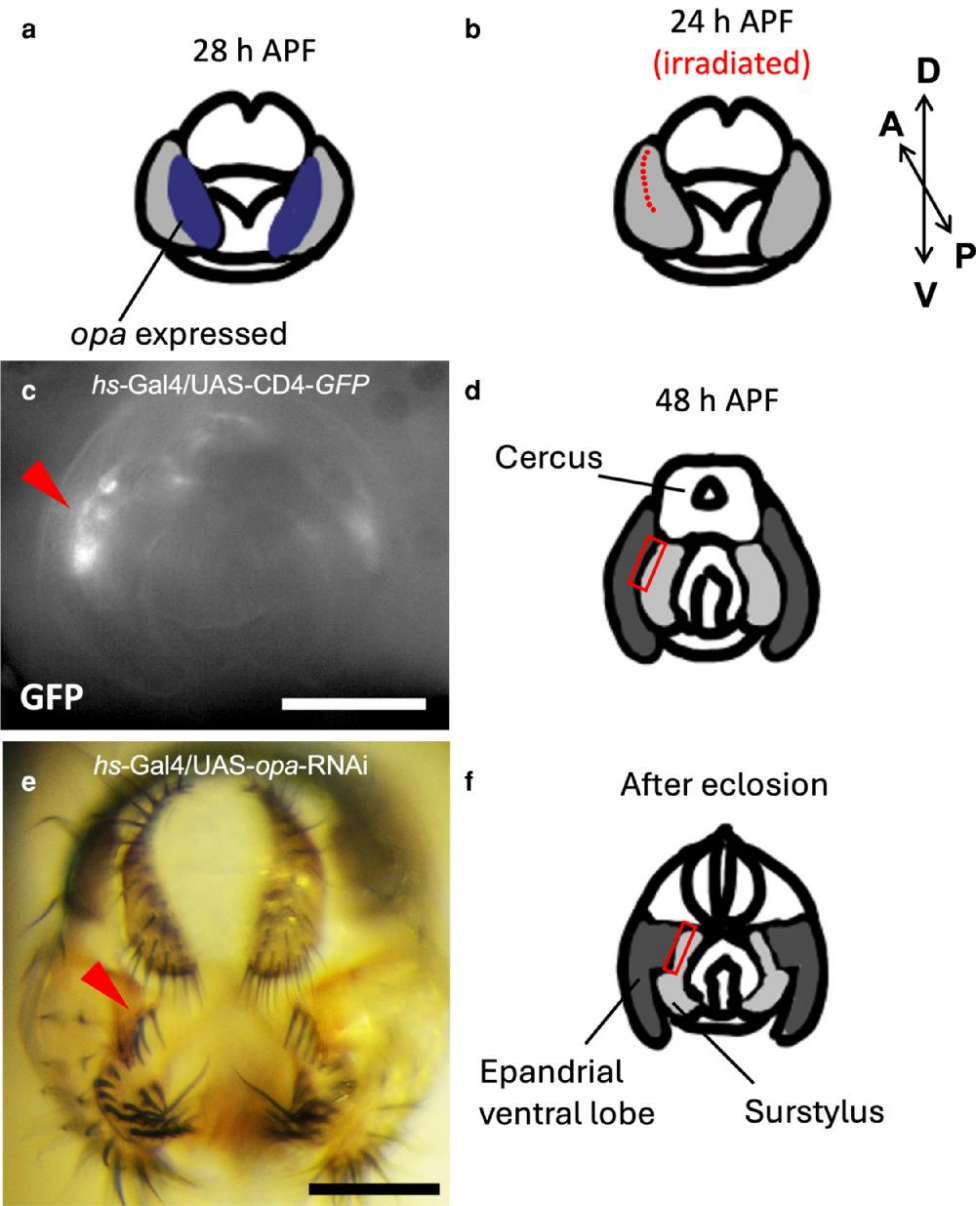
RNAi knockdown of *opa* by IR-LEGO in pupal surstylus. a) Schematic image of the pupal terminalia at 28 h APF with *opa* expression domains (indicated in blue) in accordance with Vincent et al. (2019). b) Schematic image of the pupal genitalia at 24 h APF. Light gray indicates joint epandrial ventral lobe/surstylus primordia. Red dots indicate approximate positions irradiated by an 8-mW laser for 60 s (10 times). Cleavage of the epandrial ventral lobe and surstylus initiates around this developmental time point. c) GFP signal of the pupal genitalia 1 d after infrared laser irradiation at positions shown in B of *hs-GAL4/UAS-CD4-tdGFP.* The arrowhead indicates the affected area. d) Schematic image of pupal genitalia at 48 h APF. Dark gray indicates epandrial ventral lobe, and light gray indicates surstylus. The red rectangle indicates the affected area inferred from C. e) Adult male terminalia of *hs-GAL4*/*UAS-opa-RNAi* eclosed after irradiation at positions shown in B. The arrowhead indicates the affected bristle alignment. f) Schematic image of adult genitalia after eclosion. Dark gray indicates epandrial ventral lobe, and light gray indicates surstylus. The red rectangle indicates the affected area inferred from E. Scale bars indicate 100 μm.

Motivated by the accumulating information on transcription factor expression domains in developing fly terminalia (Vincent et al. 2019), we aimed to analyze a transcription factor gene, *opa*, by knocking it down using the IR-LEGO system. This gene is expressed exclusively in the surstylus at 48 h APF and in the medial portion of the epandrial ventral lobe/surstylus domain (a single continuous epithelium) at 28 h APF (Fig. 4a), which suggests that it may play a role in identifying presumptive surstylus tissue prior to its cleavage from the epandrial ventral lobe (Vincent et al. 2019). The physical separation of the epandrial ventral lobe and surstylus begins at around 28 h APF (Urum et al. 2024).

**Fig. 5.**
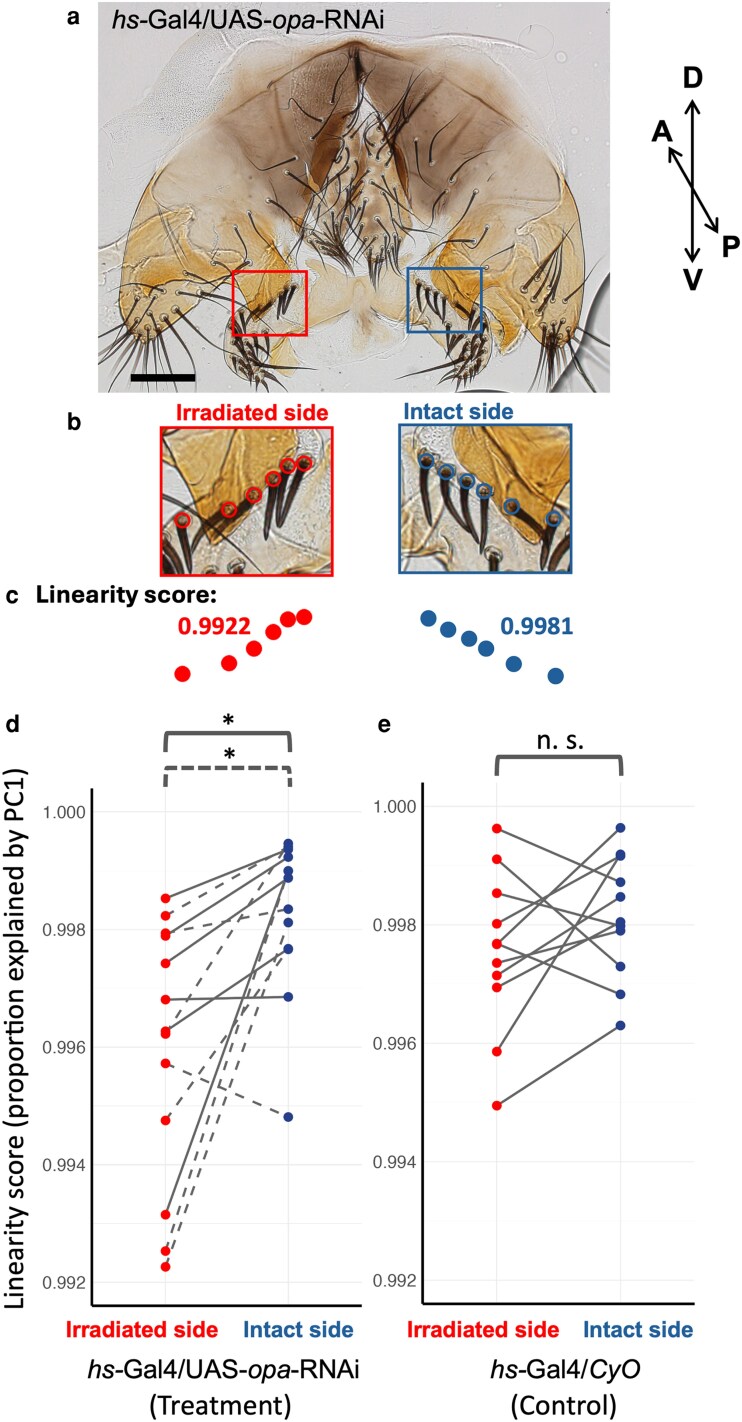
Quantification of the effect of *opa* knockdown on surstylus bristle alignment. a) A sample of the periphallic genital organs of *hs-GAL4*/*UAS-opa-RNAi* subjected to the IR-LEGO treatment shown in Fig. 4. Phallic organs were removed and mounted on a slide. The red and blue rectangles indicate the most proximal bristle rows on irradiated and intact sides, respectively. Scale bars indicate 100 μm. b) Marked positions of the bases of bristles surrounded by a socket cell. The red and blue circles represent the bristle positions of irradiated and intact sides. c) Alignments and linearity scores of the marked bristle positions of the irradiated (red) and intact (blue) sides in b. d) Linearity scores (proportions of variance explained by PC1 of the bristle position coordinates) of the irradiated and intact sides of the *hs-GAL4*/*UAS-opa-RNAi* males subjected to the IR-LEGO treatment shown in Fig. 4. The red and blue dots represent scores from the irradiated and intact sides, respectively, with gray lines connecting paired measurements from the same individuals. Two independent *UAS-opa-RNAi* strains were analyzed: v101531 (gray dashed line) and 1133R-3 (gray solid line). e) Linearity scores of the irradiated and intact sides of *hs-GAL4*/*CyO* control males of the 1133R-3 strain subjected to the same treatment as in D. The red and blue dots indicate scores from irradiated and intact sides, respectively, with gray lines connecting paired measurements from the same individuals. * indicates *P* < 0.05; n.s., not significant (*P* > 0.10) by Wilcoxon signed-rank test.

To investigate the predicted role of *opa*, we aimed at manipulating the transcript levels at the boundary region. We employed IR-LEGO and conducted the *opa* gene knockdown targeting the epandrial ventral lobe/surstylus boundary, the epithelial area close to the future cleavage site, at 24 ± 1 h APF. The 8-mW laser was applied to 10 positions for 60 s on the right or left side of the surstylus (Fig. 4b). The side with a clearer focus and a smaller depth was chosen. The response to heat shock at this timing and location was confirmed by the GFP reporter (Fig. 4, c and d). In adult flies after eclosion, we found that the alignment of the proximal bristles on the surstylus was slightly disrupted on the irradiated side (Fig. 4, e and f). To quantify this subtle change, the linearity scores of the rows of the 5 most proximal bristles on both left and right sides were obtained after dissecting the adult fly and mounting the periphallic organs on slides, as in Fig. 5a. An example of the marked positions of the bristles and the linearity scores is shown in Fig. 5, b and c.

The linearity scores were lower in the irradiated side compared to the intact side within the *hs-GAL4*/*UAS-opa-RNAi* individuals using 2 independent *UAS-opa-RNAi* strains, v101531 and 1133R-3 (Fig. 5d, paired Wilcoxon signed-rank test, *P* < 0.05, Supplementary Table 3). This indicates that the *opa* knockdown at the presumptive boundary region between the epandrial ventral lobe and surstylus perturbs the surstylus bristle alignment close to the boundary in an adult fly. To confirm that the heat damage is not the cause of the perturbation, we investigated the *hs-GAL4*/*CyO* control individuals from the 1133R-3 strain, in which there was no significant difference between the 2 sides (Fig. 5e).

To examine the broader RNAi-based targeting of *opa*, we used the genital disc-wide GAL4 driver *NP6333-GAL4* (Chatterjee et al. 2011; Tanaka et al. 2015; Hagen et al. 2019). All the progeny from the cross between *NP6333-GAL4* and the v101531 *UAS-opa-RNAi* strain died prior to eclosion at the pharate adult stage and exhibited abnormal surstyli with reduced size and severely disrupted bristle patterning (Supplementary Fig. 3). The most affected structure within the periphallic genital organ was the surstyli, suggesting that *opa* may primarily contribute to the morphogenesis of surstyli. However, the progeny of the cross between *NP6333-GAL4* and the other *UAS-opa-RNAi* strain, 1133R-3, were viable and showed only subtle changes in surstylus bristle alignment and pigmentation (Supplementary Fig. 4). The inconsistency of the RNAi effects may reflect differences in genetic background or RNAi efficacy or both.

## Discussion

Our experiment successfully employed the IR-LEGO system to knock down genes with spatial and temporal specificity in the developing genitalia of *Drosophila*. In our procedure, the target spot is irradiated for 60 s with an 8-mW infrared laser, while the previous study targeting the migrating tracheal cells of *Drosophila* embryos used a 44-mW laser for 1 s (Miao and Hayashi 2015). Many other previous studies have also employed 0.5- to 1-s irradiation (Deguchi et al. 2009; Kimura et al. 2013; Kawasumi-Kita et al. 2015), but longer durations have been applied to live cells of plants as they are immobile (Hwang et al. 2019; Tomoi et al. 2023, 2024). After checking the immobility of the pupal genital structures at APF 24 and 48 h upon infrared laser irradiation, we applied a longer irradiation duration (60 s) with lower laser power (6 to 8 mW), which was suggested in previous studies (Kamei et al. 2009; Tomoi et al. 2024) to ensure a more robust induction of heat-mediated transcription. However, pupae at later stages become mobile inside the puparia; therefore, irradiation condition may need to be adjusted.

Pupal epidermis is covered by a thin transparent pupal cuticle, and the genital primordia develop near the posterior end of the cuticle. Thus, developing genitalia has been a favorable material to conduct in vivo imaging through the lens placed outside the cuticle (Suzanne et al. 2010; Kuranaga et al. 2011; Sato et al. 2015). With the IR-LEGO system, the cells in the focal plane can be irradiated in vivo through water and aquatic tissues inside the cuticle. Since the genital tissues are not completely transparent, the cells close to the surface are the most effective target. Although IR-LEGO has a resolution of targeting single cells, those in the distant position from the surface will be difficult to irradiate with sufficient resolution. We aimed to focus on the cells at or close to the surface. Based on GFP responses in targeted cell groups at varying depths, the success rate of GFP induction was highest within a depth range of 35 to 70 μm using an 8-mW laser and declined for targets deeper than 100 μm (Fig. 2e). This likely represents a depth-related limitation for the pupal genitalia as well as other pupal tissues with comparable opacity.

The phenotypic effect of the heat-mediated manipulation of gene expression can be assessed with high sensitivity by comparing left and right sides within the same individual when 1 side is irradiated leaving the other side intact. In addition to this internal control, the potential effect of heat-induced stress responses or diffusion of the heat shock effect beyond the irradiated area should also be considered. Kamei et al. (2009) have examined the target specificity by quantifying temperature changes and GFP induction in a *C. elegans* tissue model. Under high laser power condition (33-mW, 5-s irradiation), the temperature at the focal point reached 58 °C, whereas temperature elevation was minimal at locations 20 μm away from the laser focus when the ambient temperature was 25 °C, indicating that the target range is confined to a small region. They also showed that, although higher laser power increased the proportion of cells inducing GFP compared with 11-mW irradiation, some irradiated cells appeared to be damaged at powers above 13 mW. These data indicate that heat shock responses are most strongly induced when the cell is experiencing severe heat stress. Accordingly, it is necessary to be aware that some cells can be damaged by heat or affected by the general heat shock response of the cell even when using the lower laser power applied in this study (8 mW). In embryos both with and without *hs*-*branchless*, an inhibitory effect on terminal branch formation during the dorsal tracheal development was observed, indicating that the effect was due to irradiation itself (Miao and Hayashi 2015). Likewise, irradiating samples with and without RNAi-inducing transgene would be effective in controlling for the potential effect of the heat shock response. In our study, we dissected the adults eclosed from heat-mediated induction of GFP as a control for *y* knockdown (Supplementary Figs. 1 and 2) and used *CyO* control in the case of *opa* knockdown experiment (Fig. 5d). These controls are essential for the IR-LEGO experiments.

The GAL4/UAS system has been widely used to manipulate gene expression at specific timing and space when an appropriate GAL4 driver is available. *hs*-*GAL4* has also been a useful tool to induce ectopic expression or RNAi knockdown by combining it with various transgenes with the UAS promoter (Bainton et al. 2005; Kim et al. 2018; Seong et al. 2019). Inducing heat shock ubiquitously at the whole organism level is possible by typically placing late-stage pupae or adults at 37 °C for 20 to 60 min (eg Armstrong et al. 2006; Nakayama et al. 2014). In this study, we successfully showed that the IR-LEGO heat-mediated manipulation via *hs*-*GAL4* can be performed at a confined position and timing during the genitalia morphogenesis by the *y*-*RNAi*. Compared to other genes such as *ebony* and *tan* involved in cuticle pigmentation, *y* is known to be expressed at an earlier timing during the pupal period. In the pupal wings, the transcript is most abundant at around 52 h APF (Sobala and Adler 2016) and declines while the protein becomes abundant (Riedel et al. 2011; Hinaux et al. 2018). In the abdomen and thorax, Yellow protein was detected at around 60 to 80 h APF, and expression in cells associated with bristles has also been reported (Wittkopp et al. 2002; Hinaux et al. 2018). Transcription of inverted repeat sequence of this gene at 48 h APF in our study was effective in reducing the dark pigmentation of the bristles on female and male genital organs, which coincides with these previous studies.

Among many transcription factors expressed during genital morphogenesis, *opa* is a transcription factor that is presumed to be involved in shaping the boundary between epandrial ventral lobe and surstylus (Vincent et al. 2019). These 2 structures develop from shared primordia and start separating by 28 h APF (Urum et al. 2024). *opa* marks the surstylus tissue at this time point and retains its surstylus-specific expression up to 48 h APF at least (Vincent et al. 2019). We have irradiated the boundary epithelial tissues at 24 h APF, before the cleavage begins, and found that the knockdown of *opa* at this timing and location affects the proximal bristle alignment of the surstylus (Figs. 4 and 5). Because these proximal bristles are located in the lateral surstylus, their positional alignment may have been directly affected by the *opa-RNAi* knockdown. Alternatively, the observed effects could reflect secondary consequences of altered tissue patterning. Our results do not allow us to precisely distinguish between these possibilities. Resolving this issue will require higher-resolution imaging combined with cell lineage tracking, which could potentially be achieved by further expanding this experimental system using IR-LEGO.

This phenotypic effect was consistently observed using 2 independent *UAS-opa-RNAi* strains (Fig. 5d), despite the different outcomes observed when RNAi was induced using *NP6333-GAL4*. Because RNAi induction by *NP6333-GAL4* is not restricted to the genital disc and is maintained over a longer period (Stieper et al. 2008; Tang et al. 2011), the basis for the differences in lethality and genital phenotypes remains unclear. These observations suggest that spatially and temporally confined RNAi induction could potentially reduce phenotypic variability arising from the differences in genetic background or RNAi efficacy.

The proximal half of the surstylus is attached to the epandrial ventral lobe, and the proximal bristles are located along the edge of the medial surface of the surstylus close to the protruded epandrial posterior lobe. The knockdown of *opa* in cells close to the boundary may disrupt the fate of some presumptive surstylus cells and perturb the linearity of the bristle alignment on the surstylus. There were no apparent differences in the shape or size of the posterior lobes between the irradiated and intact sides, suggesting the possibility that *opa* is primarily determining the fate of the surstylus cells and is not involved in the epandrium morphogenesis to a large extent. Further experiments to induce knockdown or ectopic expression of this gene in different cells and time using this system would elucidate its role in genital morphogenesis.

The transcription induction in our system is confined to small groups (likely to be <5) of cells (Fig. 2, b’ and d’). For targeting a larger epithelial area, irradiating multiple sites is necessary as in the case of knocking down *opa* along the epandrial ventral lobe/surstylus boundary (Fig. 4a). Even under these conditions, the phenotypic effect of *opa* knockdown using IR-LEGO was subtle compared with the clear pigmentation changes induced by *y* knockdown. Thus, a current limitation of this approach for investigating transcription factor functions is that the number of targeted cells is limited, resulting in relatively subtle phenotypic outcome. A possible way to overcome this limitation would be to shorten the irradiation time at each site by increasing the laser power and irradiate a larger number of sites. Alternatively, reducing the magnification of the lens or focusing the laser on the water immediately in front of the target may increase the effective targeting area. These approaches will require systematic optimization of parameters such as laser power, irradiation duration, and target depth, and will be important directions for future methodological development.

Although we used a custom-built device by Sigma Koki in this study, the irradiation unit of the IR-LEGO system is commercially available from Sigma Koki (Japan) or the OptoSigma group (worldwide) and can currently (as of January 2026) be used with inverted or upright microscopes from Evident or Nikon. Alternatively, a custom assembly of the system is possible using commercially available components, provided that the laser can be focused on the sample plane, as illustrated in the simplified schematic in Kamei et al. (2009). These features allow implementation of the IR-LEGO system in various configurations (eg Suzuki et al. 2013; Singhal and Shaham 2017; He et al. 2020; Tomoi et al. 2024).

In summary, we showed the potential of using the IR-LEGO system to induce heat-mediated transcription of inverted repeat sequences through the GAL4/UAS system to conduct RNAi knockdown. While many studies focus on imaginal discs, boundary formation and tissue differentiation continue to take place through the pupal period to form complex genital structures that are unique to each species. Manipulating gene expression at the focal position is certainly an attractive method without relying on the availability of transgenic strains with regulatory sequences that can target specific tissues at specific timing. Furthermore, advanced genetic tools that incorporate the heat shock promoter, such as *hs-Cre* (ie Kobayashi et al. 2013), *hs-FLP*, and *hs-Cas9*, can be tested immediately using this system.

## Supplementary Material

jkag035_Supplementary_Data

## Data Availability

All data are provided in the Supplementary Tables 1 to 3. Raw images supporting these analyses are provided as Supplementary_file_images available from Figshare at https://doi.org/10.25387/g3.28781798. Supplemental material available at G3 online.

## References

[jkag035-B1] Armstrong JD, Texada MJ, Munjaal R, Baker DA, Beckingham KM. 2006. Gravitaxis in *Drosophila melanogaster*: a forward genetic screen. Genes Brain Behav. 5:222–239. 10.1111/j.1601-183X.2005.00154.x. 16594976

[jkag035-B2] Asakawa K, Kawakami K. 2008. Targeted gene expression by the Gal4-UAS system in zebrafish. Dev Growth Differ. 50:391–399. 10.1111/J.1440-169X.2008.01044.X. 18482403

[jkag035-B3] Bainton RJ et al 2005. Moody encodes two GPCRs that regulate cocaine behaviors and blood-brain barrier permeability in *Drosophila*. Cell. 123:145–156. 10.1016/j.cell.2005.07.029. 16213219

[jkag035-B4] Boll W, Noll M. 2002. The *Drosophila* Pox neuro gene: control of male courtship behavior and fertility as revealed by a complete dissection of all enhancers. Development. 129:5667–5681. 10.1242/dev.00157. 12421707

[jkag035-B5] Brand AH, Perrimon N. 1993. Targeted gene expression as a means of altering cell fates and generating dominant phenotypes. Development (Cambridge, England). 118:401–415. 10.1242/DEV.118.2.401. 8223268

[jkag035-B6] Chatterjee SS, Uppendahl LD, Chowdhury MA, Ip PL, Siegal ML. 2011. The female-specific Doublesex isoform regulates pleiotropic transcription factors to pattern genital development in *Drosophila*. Development. 138:1099–1109. 10.1242/dev.055731. 21343364

[jkag035-B7] Deguchi T et al 2009. Infrared laser-mediated local gene induction in medaka, zebrafish and *Arabidopsis thaliana*. Dev Growth Differ. 51:769–775. 10.1111/j.1440-169X.2009.01135.x. 19843153

[jkag035-B8] Eberhard WG . 1985. Sexual selection and animal genitalia. Harvard University Press.

[jkag035-B9] Frazee SR, Masly JP. 2015. Multiple sexual selection pressures drive the rapid evolution of complex morphology in a male secondary genital structure. Ecol Evol. 5:4437–4450. 10.1002/ece3.1721. 26664690 PMC4667835

[jkag035-B10] Glassford WJ et al 2015. Co-option of an ancestral Hox-regulated network underlies a recently evolved morphological novelty. Dev Cell. 34:520–531. 10.1016/j.devcel.2015.08.005. 26343453 PMC4573913

[jkag035-B11] Gorfinkiel N, Sánchez L, Guerrero I. 1999. *Drosophila* terminalia as an appendage-like structure. Mech Dev. 86:113–123. 10.1016/S0925-4773(99)00122-7. 10446270

[jkag035-B12] Hagen JFD et al 2019. *Tartan* underlies the evolution of *Drosophila* male genital morphology. Proc Natl Acad Sci U S A. 116:19025–19030. 10.1073/pnas.1909829116. 31484761 PMC6754542

[jkag035-B13] Halpern ME et al 2008. Gal4/UAS transgenic tools and their application to zebrafish. Zebrafish. 5:97–110. 10.1089/ZEB.2008.0530. 18554173 PMC6469517

[jkag035-B14] Hasugata R et al 2018. Infrared laser-mediated gene induction at the single-cell level in the regenerating tail of *Xenopus laevis* tadpoles. Cold Spring Harb Protoc. 2018:pdb.prot101014. 10.1101/pdb.prot101014. 29769391

[jkag035-B15] He S et al 2020. In vivo single-cell lineage tracing in zebrafish using high-resolution infrared laser-mediated gene induction microscopy. eLife. 9:e52024. 10.7554/eLife.52024. 31904340 PMC7018510

[jkag035-B16] Hinaux H et al 2018. Revisiting the developmental and cellular role of the pigmentation gene *yellow* in *Drosophila* using a tagged allele. Dev Biol. 438:111–123. 10.1016/j.ydbio.2018.04.003. 29634916

[jkag035-B17] Hosken DJ, Stockley P. 2004. Sexual selection and genital evolution. Trends Ecol Evol. 19:87–93. 10.1016/J.TREE.2003.11.012. 16701234

[jkag035-B18] Hwang D et al 2019. Development of a heat-inducible gene expression system using female gametophytes of *Arabidopsis thaliana*. Plant Cell Physiol. 60:2564–2572. 10.1093/pcp/pcz148. 31359050

[jkag035-B19] Kamei Y et al 2009. Infrared laser-mediated gene induction in targeted single cells in vivo. Nat Methods. 6:79–81. 10.1038/nmeth.1278. 19079252

[jkag035-B20] Kawasumi-Kita A et al 2015. Application of local gene induction by infrared laser-mediated microscope and temperature stimulator to amphibian regeneration study. Dev Growth Differ. 57:601–613. 10.1111/dgd.12241. 26510480

[jkag035-B21] Keisman EL, Baker BS. 2001. The *Drosophila* sex determination hierarchy modulates *wingless* and *decapentaplegic* signaling to deploy *dachshund* sex-specifically in the genital imaginal disc. Development (Cambridge, England). 128:1643–1656. 10.1242/DEV.128.9.1643. 11290302

[jkag035-B22] Kim JH et al 2018. RNA interference validation of detoxification genes involved in ivermectin tolerance in *Drosophila melanogaster*. Insect Mol Biol. 27:651–660. 10.1111/imb.12512. 29888824

[jkag035-B23] Kimura E et al 2013. Application of infrared laser to the zebrafish vascular system: gene induction, tracing, and ablation of single endothelial cells. Arterioscler Thromb Vasc Biol. 33:1264–1270. 10.1161/ATVBAHA.112.300602. 23539214

[jkag035-B24] Kobayashi K, Kamei Y, Kinoshita M, Czerny T, Tanaka M. 2013. A heat-inducible CRE/LOXP gene induction system in medaka. Genesis. 51:59–67. 10.1002/dvg.22348. 23019184

[jkag035-B25] Kuranaga E et al 2011. Apoptosis controls the speed of looping morphogenesis in *Drosophila* male terminalia. Development. 138:1493–1499. 10.1242/dev.058958. 21389055

[jkag035-B26] McQueen EW et al 2022. A standardized nomenclature and atlas of the female terminalia of *Drosophila melanogaster*. Fly 16:128–151. 10.1080/19336934.2022.2058309. 35575031 PMC9116418

[jkag035-B27] Miao G, Hayashi S. 2015. Manipulation of gene expression by infrared laser heat shock and its application to the study of tracheal development in *Drosophila*. Dev Dyn. 244:479–487. 10.1002/dvdy.24192. 25258210

[jkag035-B28] Nagy O et al 2018. Correlated evolution of two copulatory organs via a single *cis*-regulatory nucleotide change. Curr Biol. 28:3450–3457.e13. 10.1016/j.cub.2018.08.047. 30344115 PMC7385753

[jkag035-B29] Nakayama M et al 2014. A gain-of-function screen to identify genes that reduce lifespan in the adult of *Drosophila melanogaster*. BMC Genet. 15:46. 10.1186/1471-2156-15-46. 24739137 PMC4021436

[jkag035-B30] Okuyama T et al 2013. Controlled Cre/loxP site-specific recombination in the developing brain in medaka fish, *Oryzias latipes*. PLoS One. 8:e66597. 10.1371/JOURNAL.PONE.0066597. 23825546 PMC3692484

[jkag035-B31] Pedregosa F, et al 2011. Scikit-learn: machine learning in Python. J Mach Learn Res. 12:2825–2830. https://dl.acm.org/doi/10.5555/1953048.2078195.

[jkag035-B32] Rice G et al 2019. A standardized nomenclature and atlas of the male terminalia of *Drosophila melanogaster*. Fly 13:51–64. 10.1080/19336934.2019.1653733. 31401934 PMC6988887

[jkag035-B33] Ridgway AM, Hood EJ, Jimenez JF, Nunes MDS, McGregor AP. 2024. *Sox21b* underlies the rapid diversification of a novel male genital structure between *Drosophila* species. Curr Biol. 34:1114–1121.e7. 10.1016/j.cub.2024.01.022. 38309269

[jkag035-B34] Riedel F, Vorkel D, Eaton S. 2011. Megalin-dependent Yellow endocytosis restricts melanization in the *Drosophila* cuticle. Development. 138:149–158. 10.1242/dev.056309. 21138977

[jkag035-B35] Sato K et al 2015. Left-right asymmetric cell intercalation drives directional collective cell movement in epithelial morphogenesis. Nat Commun. 6:10074. 10.1038/NCOMMS10074. 26656655 PMC4682055

[jkag035-B36] Schindelin J, et al 2012. Fiji: an open-source platform for biological-image analysis. Nat Methods. 9:676–682. 10.1038/nmeth.2019. 22743772 PMC3855844

[jkag035-B37] Seong KM, Coates BS, Pittendrigh BR. 2019. Cytochrome P450s *Cyp4p1* and *Cyp4p2* associated with the DDT tolerance in the *Drosophila melanogaster* strain 91-R. Pestic Biochem Physiol. 159:136–143. 10.1016/j.pestbp.2019.06.008. 31400775

[jkag035-B38] Shimada A et al 2013. Trunk exoskeleton in teleosts is mesodermal in origin. Nat Commun. 4:1639. 10.1038/NCOMMS2643. 23535660 PMC3615485

[jkag035-B39] Shimizu R et al 2024. Spatiotemporal control of transgene expression using an infrared laser in the crustacean *Daphnia magna*. Sci Rep. 14:1–8. 10.1038/s41598-024-77458-8. 39465323 PMC11514169

[jkag035-B40] Simmons LW . 2014. Sexual selection and genital evolution. Aust Entomol. 53:1–17. 10.1111/aen.12053.

[jkag035-B41] Singhal A, Shaham S. 2017. Infrared laser-induced gene expression for tracking development and function of single *C. elegans* embryonic neurons. Nat Commun. 8:14100. 10.1038/ncomms14100. 28098184 PMC5253673

[jkag035-B42] Smith SJ, Davidson LA, Rebeiz M. 2020. Evolutionary expansion of apical extracellular matrix is required for the elongation of cells in a novel structure. eLife. 9:e55965. 10.7554/eLife.55965. 32338602 PMC7266619

[jkag035-B43] Sobala LF, Adler PN. 2016. The gene expression program for the formation of wing cuticle in *Drosophila*. PLoS Genet. 12:e1006100. 10.1371/journal.pgen.1006100. 27232182 PMC4883753

[jkag035-B44] Stieper BC, Kupershtok M, Driscoll MV, Shingleton AW. 2008. Imaginal discs regulate developmental timing in Drosophila melanogaster. Dev Biol. 321:18–26. 10.1016/j.ydbio.2008.05.556. 18632097

[jkag035-B45] Suzanne M et al 2010. Coupling of apoptosis and L/R patterning controls stepwise organ looping. Curr Biol. 20:1773–1778. 10.1016/j.cub.2010.08.056. 20832313 PMC4516037

[jkag035-B46] Suzuki M et al 2022. Mosaic gene expression analysis of semaphorin–plexin interactions in *Caenorhabditis elegans* using the IR-LEGO single-cell gene induction system. Dev Growth Differ. 64:230–242. 10.1111/dgd.12793. 35596523

[jkag035-B47] Suzuki M, Toyoda N, Shimojou M, Takagi S. 2013. Infrared laser-induced gene expression in targeted single cells of *Caenorhabditis elegans*. Dev Growth Differ. 55:454–461. 10.1111/dgd.12061. 23614811

[jkag035-B48] Tanaka KM et al 2015. Genetic architecture and functional characterization of genes underlying the rapid diversification of male external genitalia between *Drosophila simulans* and *Drosophila mauritiana*. Genetics. 200:357–369. 10.1534/genetics.114.174045. 25783699 PMC4423377

[jkag035-B49] Tang HY, Smith-Caldas MS, Driscoll MV, Salhadar S, Shingleton AW. 2011. FOXO regulates organ-specific phenotypic plasticity in *Drosophila*. PLoS Genet. 7:e1002373. 10.1371/journal.pgen.1002373. 22102829 PMC3213149

[jkag035-B50] Tomoi T et al 2023. Targeted single-cell gene induction by optimizing the dually regulated CRE/loxP system by a newly defined heat-shock promoter and the steroid hormone in *Arabidopsis thaliana*. Front Plant Sci. 14:1171531. 10.3389/FPLS.2023.1171531. 37351202 PMC10283073

[jkag035-B51] Tomoi T et al 2024. Infrared laser-induced gene expression in single cells characterized by quantitative imaging in *Physcomitrium patens*. Commun Biol. 7:1448. 10.1038/s42003-024-07141-1. 39506095 PMC11541703

[jkag035-B52] Urum A et al 2024. A developmental atlas of male terminalia across twelve species of *Drosophila*. Front Cell Dev Biol. 12:1349275. 10.3389/fcell.2024.1349275. 38487271 PMC10937369

[jkag035-B53] Urum M, Preger-Ben Noon E. 2025. The developmental and genetic basis of male genitalia evolution in Drosophilids. Curr Opin Insect Sci. 68:101335. 10.1016/j.cois.2025.101335. 39880363

[jkag035-B54] Vincent BJ et al 2019. An atlas of transcription factors expressed in male pupal terminalia of *Drosophila melanogaster*. G3 (Bethesda). 9:3961–3972. 10.1534/g3.119.400788. 31619460 PMC6893207

[jkag035-B55] Wittkopp PJ, True JR, Carroll SB. 2002. Reciprocal functions of the *Drosophila yellow* and *ebony* proteins in the development and evolution of pigment patterns. Development (Cambridge, England). 129:1849–1858. 10.1242/DEV.129.8.1849. 11934851

