# Supplementary material for: Heat-mediated manipulation of gene expression by IR-LEGO in the developing genitalia in Drosophila
Source: G3 (Bethesda). 2026 Feb 12;16(4):jkag035. doi: 10.1093/g3journal/jkag035 (PMC13042308; doi:10.1093/g3journal/jkag035)
Supplement: jkag035_Supplementary_Data [file jkag035_supplementary_data.zip › Supplementary_Fig._2_G3-2025-406178.pdf]

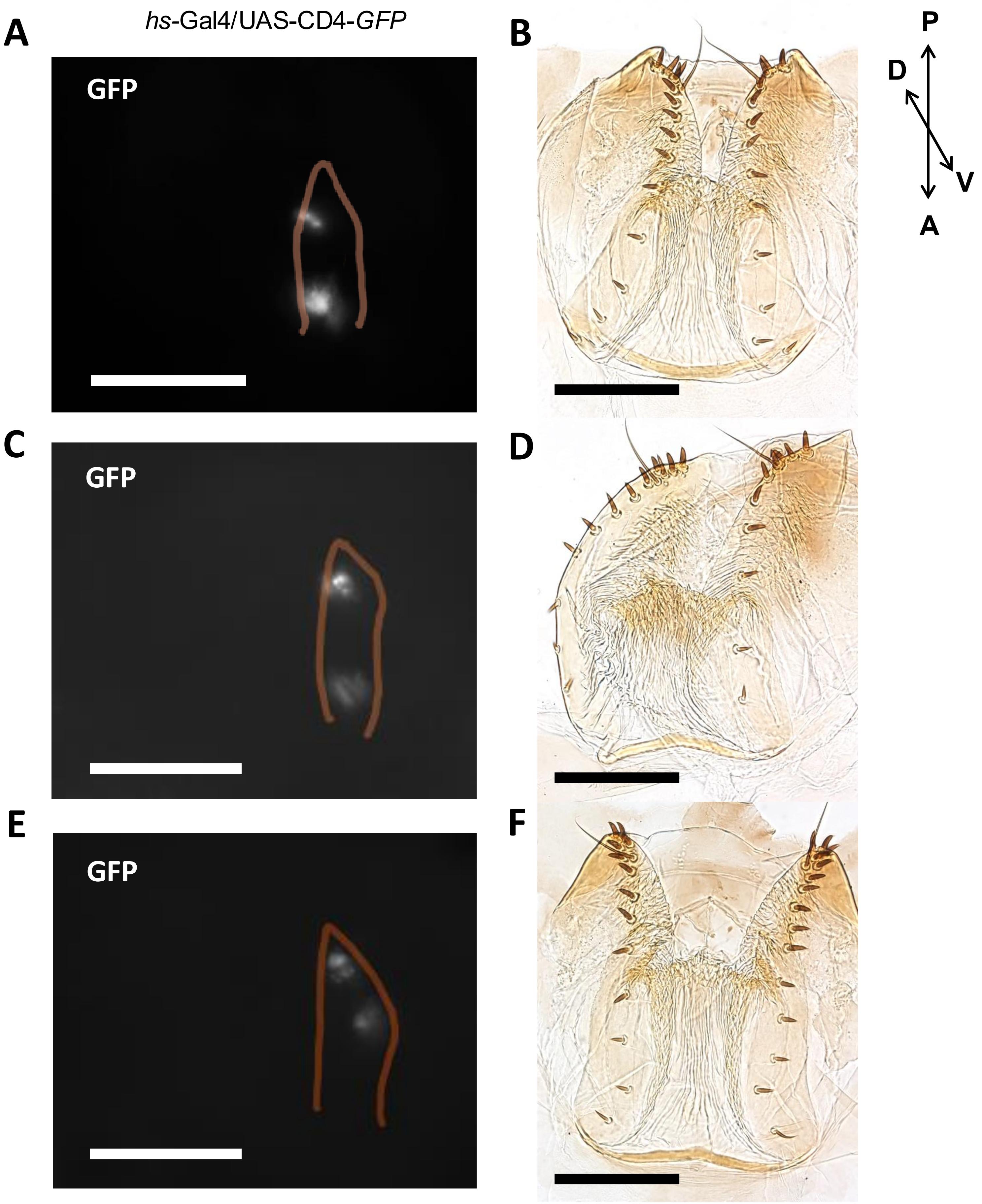

Supplementary Fig. 2 Hypogynial valve images of sampled individuals subjected to IR-LEGO in Figure 2. (A)(C)(E) GFP signals observed one day after irradiation at 48 ± 1 h APF. Outlines of the irradiated valves are shown. (B)(D)(F) Mounted hypogynial valve images of the same individuals after eclosion as in (A)(C)(E), respectively, indicating no notable changes in bristle color. Scale bars indicate 100 μm.

Supplementary Figure 2
