# Supplementary material for: Heat-mediated manipulation of gene expression by IR-LEGO in the developing genitalia in Drosophila
Source: G3 (Bethesda). 2026 Feb 12;16(4):jkag035. doi: 10.1093/g3journal/jkag035 (PMC13042308; doi:10.1093/g3journal/jkag035)
Supplement: jkag035_Supplementary_Data [file jkag035_supplementary_data.zip › Supplementary_Fig._3_G3-2025-406178.pdf]

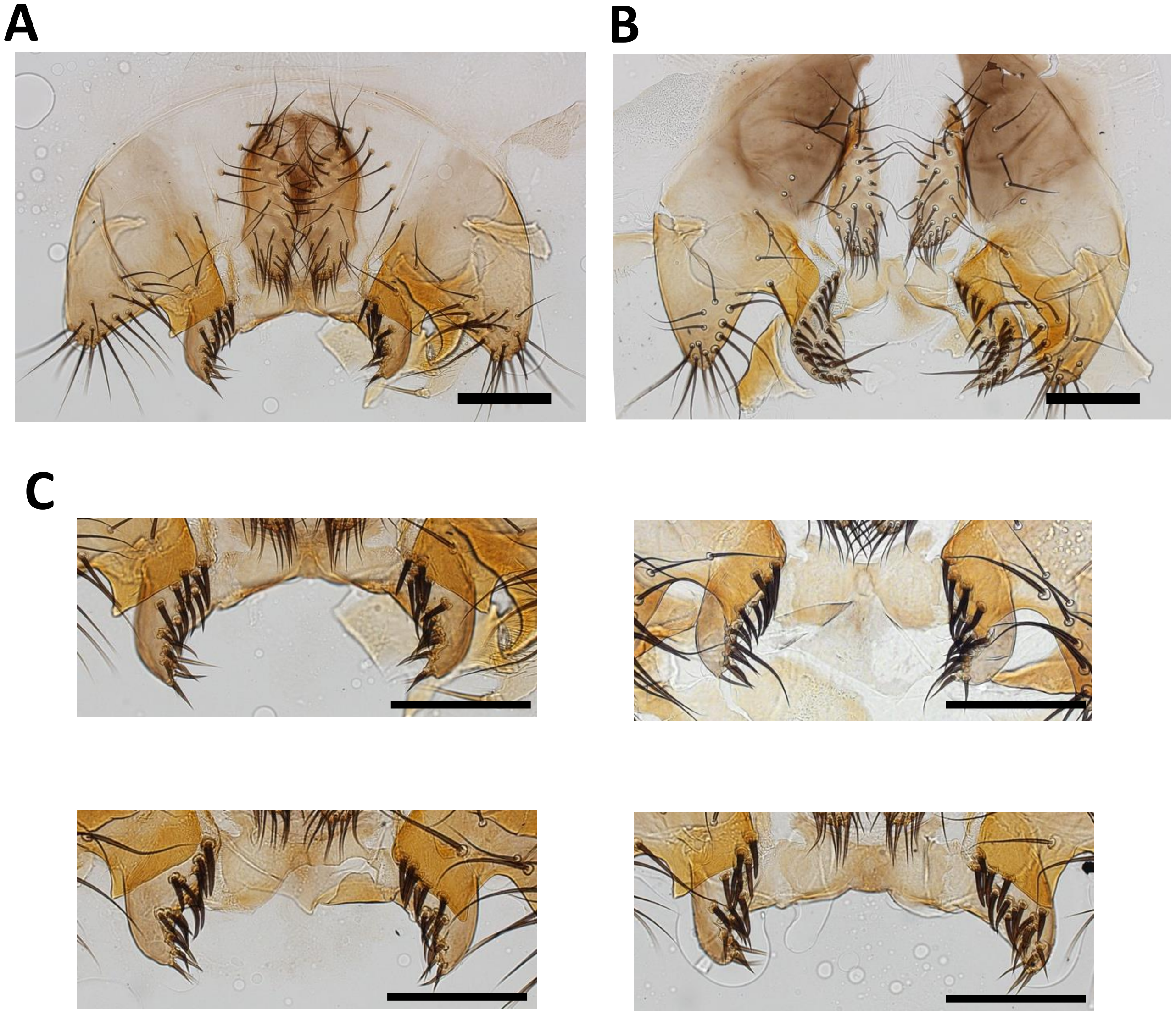

Supplementary Fig. 3 Surstylus morphology in *NP6333-Gal4/UAS-opa-RNAi* (v101531) males. (A) Periphalic genital organ of a pharate adult that died prior to eclosion, showing reduced surstylus size and severely disrupted bristle patterning. (B) *UAS-opa-RNAi* (v101531) male as a control. (C) Higher-magnification images of surstyli from pharate adults that died prior to eclosion. Scale bars indicate 100 μm.
